# Outcome of primary rhegmatogenous retinal detachment using microincision vitrectomy and sutureless wide-angle viewing systems

**DOI:** 10.1186/s12886-019-1238-3

**Published:** 2019-11-19

**Authors:** Chun-Ting Lai, Wei-Hsun Kung, Chun-Ju Lin, Huan-Sheng Chen, Henry Bair, Jane-Ming Lin, Wen-Lu Chen, Peng-Tai Tien, Yi-Yu Tsai

**Affiliations:** 1 Department of Ophthalmology, China Medical University Hospital, China Medical University, 2 Yuh-Der Road, Taichung City, Taiwan 40447; 2 School of Medicine, College of Medicine, China Medical University, Taichung, Taiwan; 3 Department of Optometry, Asia University, Taichung, Taiwan; 4 An-Shin Dialysis Center, NephroCare Ltd., Fresenius Medical Care, Taichung, Taiwan; 5 Stanford University School of Medicine, Stanford, USA; 6 Graduate Institute of Clinical Medical Science, China Medical University, Taichung, Taiwan

**Keywords:** Non-contact wide angle viewing system, Sutureless, Micro-incision vitrectomy surgery, Rhegmatogenous retinal detachment

## Abstract

**Background:**

To evaluate the efficacy of micro-incision vitrectomy surgery (MIVS) using Lumera and Resight non-contact sutureless wide-angle viewing systems (WAVS) for primary rhegmatogenous retinal detachment (RRD), and to analyze the anatomical and visual outcomes.

**Methods:**

The retrospective, non-comparative, interventional case series reported here was conducted from June 2014 through November 2016. Enrolled patients presented with primary RRD and received MIVS with/without cryopexy by one surgeon using the Lumera and Resight non-contact sutureless WAVS. All patients were followed-up for a minimum of 12 months. Variables collected included patient demographics, best-corrected visual acuity, and macular status. The number and position of retinal break(s), and the use of cryopexy, were also recorded. Outcome measures included operative time, single-operation anatomical success rate, final anatomical success rate, recurrent rate, postoperative best-corrected visual acuity, and surgical complications. The end points were operative time, anatomical outcome, and functional outcome.

**Results:**

In total, 110 eyes from 110 patients (68 men and 42 women) were treated. Of these, 103 (93%) eyes were reattached after primary vitrectomy. One hundred ten eyes (100%) reached final anatomical success. The mean operative time was 50.55 min. Multivariate analyses were performed with best model selection principle based on general linear model by Akaike Information Criteria for detecting possible factors related to operation time, and with multivariate logistic regression analysis for revealing probable clinical parameters which might influence the anatomical outcome after first operation and final visual outcome. Intraoperative cryopexy and multiple breaks increased operative time significantly. More favorable BCVA was significantly correlated with shorter operation time and the preoperative macula-on status. Multivariate logistic regression on the group of patients who have received the cataract surgery revealed that the pre-operative BCVA is a significant factor which can predict the visual outcome after MIVS.

**Conclusions:**

The outcome of primary RRD repaired by MIVS using the Lumera and Resight sutureless WAVS was not inferior to any other published method. This instrument combination resulted in a relatively rapid and comfortable procedure without serious postoperative complications. Cryopexy and multiple breaks affected operative time significantly. Shorter operative times and preoperative macula-on status are associated with better final visual outcomes.

## Background

A rhegmatogenous retinal detachment (RRD) occurs when at least one retinal tear allows vitreous humor to penetrate into the subretinal space and separate the neurosensory retina from the underlying retinal pigment epithelium. RRD incidence has been reported to be between 6.3 and 17.9 per 100,000 population and demonstrates significant geographical variation [1]. Retinal specialists have long sought to improve methods for repairing the detached retina successfully while causing less damage. The developments of scleral buckling techniques, laser, and intraocular gases have been critical for improved outcomes in the repair of RRD [2–4]. The first pars plana vitrectomy (PPV), which allows vitreous removal via a closed-system vitreous infusion section cutter, was performed in 1972 [5, 6]. Since then, the instrumentation has evolved from the original 17-gauge cutter to micro-incision vitrectomy surgery (MIVS) [6, 7]. Smaller sutureless sclerotomy wounds result in less postoperative inflammation, greater postoperative patient comfort, and possibly faster postoperative visual recovery [8, 9]. On the other hand, innovations to the viewing system have included the contact lens and noncontact lens wide angle viewing systems (WAVS). The RESIGHT WAVS contains two fixated lenses (128D and 60D) that could be rotated into the light beam easily. Noncontact sutureless WAVS, unlike contact WAVS, do not require either sutures or the aid of skilled assistants to hold the lens in place during surgery [10, 11]. However, the surgeon has to adjust the focus and field of view more frequently during the operation when using noncontact sutureless WAVS than when using contact WAVS. Therefore, whether the noncontact sutureless WAVS shorten the operation is a matter of debate. In Taiwan, retinal detachment is very common, possibly due to the high incidence rate of myopia. The number of patients requiring surgery exceed the capacity of hospitals that can perform pars plana vitrectomy. In order to arrange PPV as soon as possible, a safe and faster method of operation is necessary for these patients. To our best knowledge, there have been few publications reporting the operation time of MIVS using noncontact sutureless WAVS. In the present study, we therefore set out to assess the operation time, analyze influencing factors, and offer a compatible data for MIVS using noncontact sutureless WAVS. In addition, we recorded and discuss the anatomical and visual outcomes.

## Methods

**Fig. 1:**
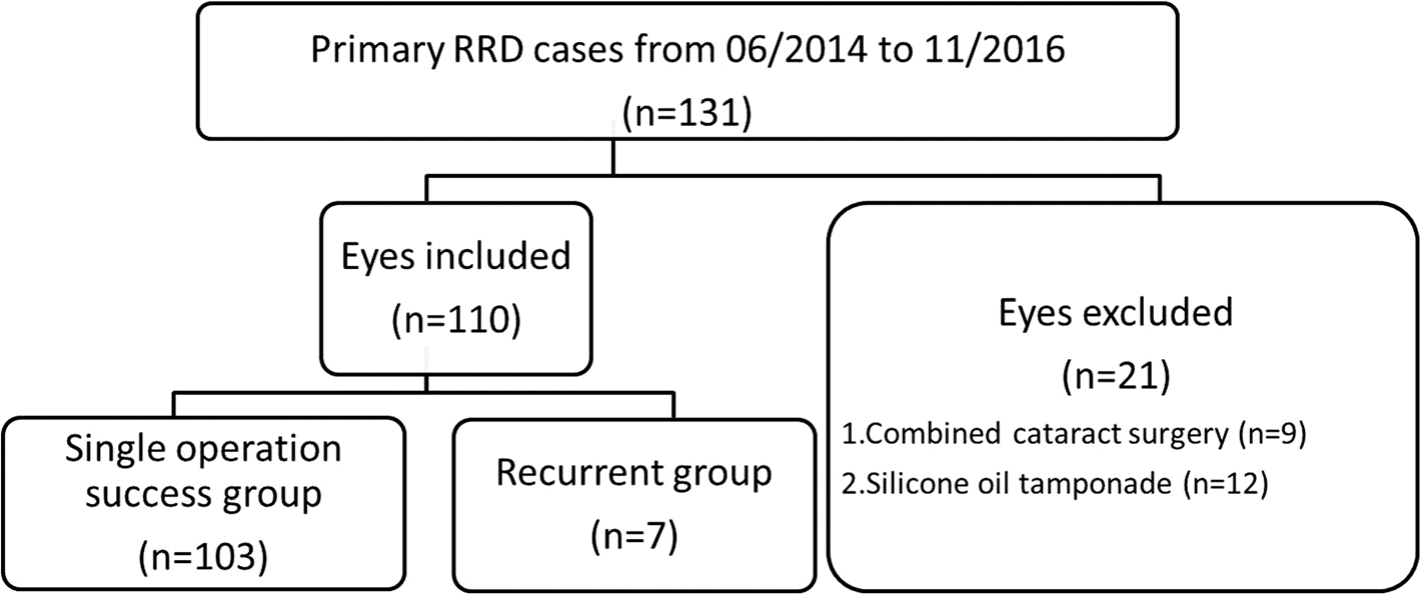
The concise inclusion and exclusion criteria of this study

This is a retrospective, comparative, interventional case series. The study protocol was conducted according to the principles described in the Declaration of Helsinki, and institutional review board/ethics committee approval was obtained. All patients provided written informed consent. One hundred ten eyes from 110 patients with a minimum of 12-month follow-up were collected from June 2014 through November 2016 at the Department of Ophthalmology, China Medical University Hospital, China Medical University. All patients enrolled in the study presented with primary RRD and received 23- or 25-gauge transconjunctival sutureless MIVS, performed by one surgeon (Dr. CJ Lin). Patients having undergone pneumatic retinopexy, vitrectomy, or scleral buckling were not included in our study. Patients with macular holes, dense cataracts that required a combined cataract operation, or who required the use of silicone oil as tamponade during operation were excluded (Fig. 1).

All surgeries were performed after administration of retrobulbar anesthesia. The trocar and infusion cannula were inserted. 25-gauge MIVS without scleral buckling was performed using the Alcon Constellation vitrectomy system (Ft Worth, TX, USA) and Lumera 700 and Resight (Carl Zeiss Meditec) noncontact type sutureless WAVS. A 23-gauge 6 mm trocar was only used for infusion cannula in patients with hypotony. Core and peripheral vitrectomy were performed with careful attention directed toward relieving all tractional forces on the peripheral retina and on all identified retinal breaks, which were marked with endocautery. Intravitreal triamcinolone was used in all cases to identify and remove the vitreous and epiretinal membrane as completely as possible. Scleral depression was performed through the noncontact sutureless WAVS to check for possible iatrogenic breaks. Fluid–air exchange with a soft-tip extrusion needle and active suction was used to drain subretinal and posterior pole fluid. Endolaser retinopexy was performed both before and after the fluid–air exchange. Transconjunctival cryopexy was used in cases of anterior retinal breaks. Internal limiting membrane peeling was not performed in any of the surgeries. Nonexpansile mixtures of either perfluoropropane (C3F8) or sulfur hexafluoride (SF6) were used in all cases. At the conclusion of each procedure, Levofloxacin eyedrops were placed on the surface of the eye followed by a patch and shield. Patients were instructed in either face-down or one-sided post-operative positioning for 14 days.

Variables collected for the study include patient demographics, preoperative best-corrected visual acuity (BCVA), intraocular pressure (IOP), lens status, and macular status. Operation records retrospectively reviewed include the number of retinal breaks noted after scleral depression, the use of cryopexy, the type of gas tamponade used, and the operation time. The postoperative IOP and the postoperative final BCVA, defined as the BCVA during follow up in patients (or in the cases of patients who subsequently underwent cataract surgery, the BCVA after the latter surgery), were also recorded.

Outcome measures included the average operation time, single-operation anatomical success rate, final anatomical success rate, postoperative BCVA, and surgical complications. All analyses were carried out using SPSS statistics 22.0. *P* < 0.05 was considered statistically significant. Multivariate analyses were performed with best model selection principle based on general linear model by Akaike Information Criteria (AIC) for detecting possible factors related to operation time, and with multivariate logistic regression analysis for revealing probable clinical parameters which might influence the anatomical outcome after first operation and final visual outcome.

## Results

### Baseline characteristics and intraoperative management

Out of 110 patients initially surveyed for the study, 110 eyes met the inclusion criteria. The median age of the patients was 54.77 years. Of the 110 cases, 68 (61.8%) were male and 42 (38.2%) were female. The mean preoperative BCVA was 20/224 (1.05 logMAR), and the mean preoperative IOP was 13.42 mmHg. Of the 110 eyes, 98 (89%) were phakic and the remaining (11%) were pseudophakic. The macula was attached preoperatively in 41 (37.2%) of 110 eyes, while macula detachment was present in the remaining eyes (62.8%). During the operation, single breaks were noted in 39 (35.5%) eyes, and multiple retinal breaks were found in 71 (64.5%) eyes. Transconjunctival cryopexy was applied adjuvant to laser on 40 (36.4%) eyes. C3F8 was used as gas tamponade after MIVS in 79 (71.8%) eyes, while SF6 was used in 31 (28.2%) eyes.

### Operation time

The mean operation time of MIVS using the Lumera and Resight non-contact sutureless WAVS for primary RRD in our study was 50.55 min. Possible factors affecting the operation time are summarized in Table 1. The use of adjuvant cryopexy and RRDs with multiple breaks increased the operation time significantly (*p* < 0.05). The age of patients, preoperative lens status, and the presence of macular detachment did not affect the operation time. This result was confirmed by multivariate analysis. With general linear model technique, cryopexy and multiple breaks were only two significant factors kept in the final model which influenced operation time (AIC 694.5, adjusted R-Square 0.124 for final model, operation time was 8.29 min longer in cryopexy group when compared to non-cryopexy group, 3.17 min longer in patients with multiple breaks when compared to single-break patients, Table 4a).

**Table 1:** Potential Factors Affecting Operation Time

Variable	Number of Cases	Mean operation time (mins)	*P* value	Methods
Age				
Age ≤ 50	33	54.79	0.051	T test
Age > 50	77	48.74		
Total	110		0.376	Regression
Cryopexy				
Not performed	70	46.61	< 0.001	T test
Performed	40	57.45		
Number of breaks				
1	39	46.31	0.026	T test
> 1	71	52.89		
Total			< 0.001	Regression
Lens status				
Phakic	92	50.34	0.663	T test
Pseudophakic	12	55.33		
Macula status				
Macula on	41	51.12	0.760	T test
Macula off	69	50.22		

### Anatomical outcome

The anatomic success rate after a single operation was 103/110 (93.6%). Seven (6.4%) eyes required one additional reoperation, and the final anatomical success rate was 100%. The 103 eyes with successfully reattached retinas after a single operation were compared with the 7 eyes with redetached retinas during the follow-up period, with respect to preoperative variables and surgical factors, including age, BCVA, IOP, refraction, lens status, macular status, the use of adjuvant cryopexy, the type of gas tamponade used, the mean operation time, and the postoperative IOP. The results are summarized in Table 2. None of the factors mentioned significantly influenced the single-operation success rate in this study. Even after multivariate analysis with logistic regression, the clinical parameters as listed in Table 2 still did not show any significant impact on the single-operation anatomical outcome. Among the 7 eyes with redetached retinas during the follow-up period, 5 eyes had more than 2 breaks (up to 8) and at least one break located inferiorly.

**Table 2:**
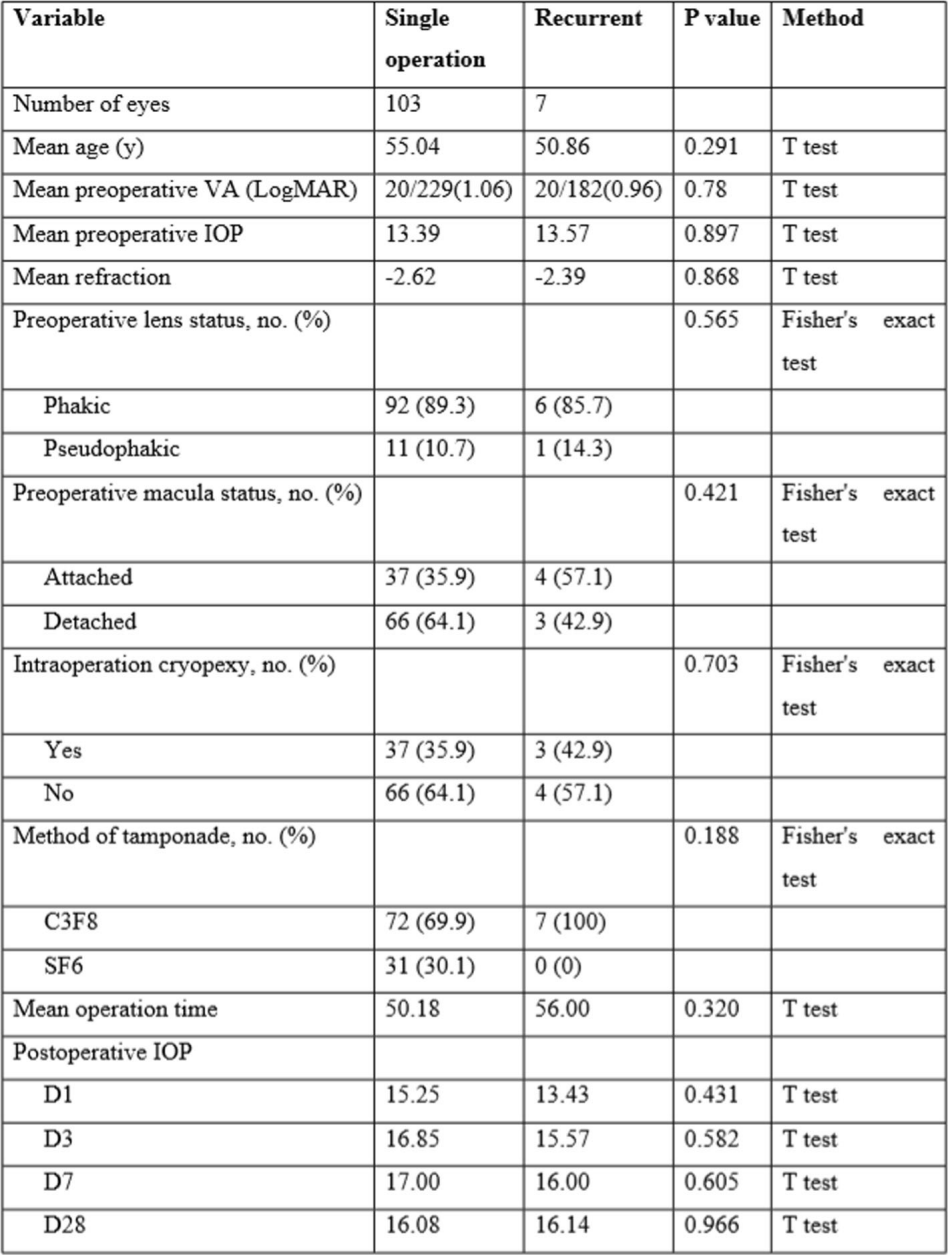
Potential Factors Affecting Anatomical Outcomes

### Visual outcome

Except for two eyes without records of postoperative BCVA, the mean BCVA improved significantly from 20/229 (1.059 logMAR) to 20/45 (0.354 logMAR) (*p* < 0.001, paired T test) for all other 108 eyes enrolled in the study. 73% (79/108) of eyes had better BCVA after MIVS, and 27% (29/108) of eyes had worse or stable BCVA. Among the 29 eyes without improvement of BCVA, 15 had not undergone cataract operation. Using ‘BCVA improved or not’ as dependent variable and all the possible clinical relevant factors (parameters in Tables 2 and 3) as independent variables, multivariate logistic regression was performed in order to reveal any possible factors which might predict the improvement of BCVA compared to pre-operative level. Pre-operative BCVA was the only significant factor found in the final model. Worse pre-operative BCVA predicted higher rate of BCVA improvement after operation (for every 0.1 logMAR increase of pre-operative BCVA, the odds of successful operation will increase 18.8%, c statistics = 0.90, Table 4b).

**Table 3:**
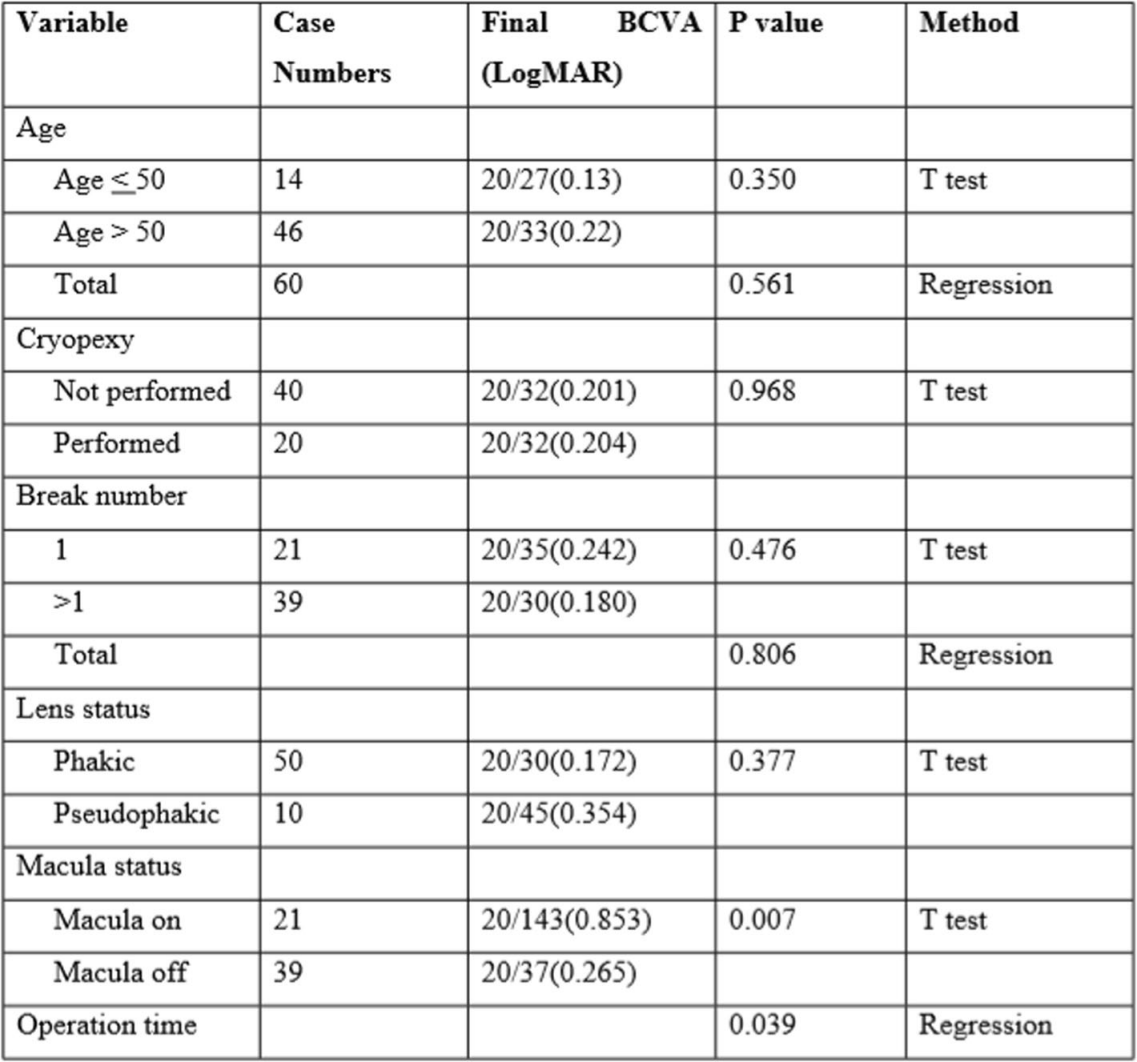
Potential Factors Affecting the Final BCVA

**Table 4:** Multivariate analysis. a) Model of prediction for operation time by general linear model (GLM). b) Model of prediction for visual acuity improvement by multivariate logistic regression. c) Model of prediction for visual acuity improvement by multivariate logistic regression only in group of patients who have received cataract surgery

a)							
Effect		Estimate	R-Square	Adjusted R-Square	AIC	F Value	Pr > F
Intercept		45.49	0	0	707.2	0	1
Use of Cryopexy		8.29	0.089	0.081	698.9	10.61	0.0015
Multiple Breaks		3.17	0.14	0.124	694.5	6.38	0.013
b)							
Parameter	DF	Estimate	Standard Error	Wald Chi-Square	Pr > ChiSq	Odds Ratio (95% CI)	C statistics (AUC)
Intercept	1	−0.61	0.42	2.05	0.1522		
Pre-operative VA (logMar)	1	2.93	0.85	11.78	0.0006	18.8 (3.52–100.4)	
							0.90
c)							
Parameter	DF	Estimate	Standard Error	Wald Chi-Square	Pr > ChiSq	Odds Ratio (95% CI)	C statistics (AUC)
Intercept	1	−0.32	0.58	0.31	0.5766		
Pre-operative VA (logMar)	1	3.42	1.55	4.84	0.0278	30.4 (1.45–637.2)	
							0.91

*AIC* Akaike Information Criteria, *Pr* Probability

*DF* Degree of freedom, *AUC* Area under curve

*DF* Degree of freedom, *AUC* Area under curve

Post-PPV cataract formation can considerably decrease vision even the function of retina is well improved. In order to identify the factors affecting visual outcome after the single operation, we further excluded the preoperative phakic eyes that did not undergo cataract surgery after MIVS, as well as the eyes with recurrent RRD. Sixty eyes remained, and visual outcome was analyzed with respect to preoperative measures and surgical factors. The results are summarized in Table 3. More favorable BCVA was significantly correlated with shorter operation times (*p* < 0.05) and the preoperative macula-on status (p < 0.05). Focusing on improvement of BCVA, multivariate logistic regression on the group of patients who have received the cataract surgery revealed that the pre-operative BCVA is a significant factor which can predict the visual outcome after MIVS (for every 0.1 logMAR increase of pre-operative VA, the odds of successful operation will increase 30.4%, c statistics = 0.91, Table 4c).

### Complications

No expulsive choroidal hemorrhages, hypotony or endophthalmitis occurred during or after any of the operations.

## Discussion

In this study, we report the results of a retrospective analysis on the repair of primary RRD through sutureless MIVS with the use of a noncontact sutureless WAVS. The noncontact sutureless WAVS have several advantages over the hand-held irrigating lens systems or the self-irrigating fixed contact lens retaining ring system [12]. The noncontact sutureless WAVS does not require an experienced assistant to hold the lens in place, thus the risk of excessive pressure on the cornea, causing corneal epithelial defect or edema, is avoided. Furthermore, fixation sutures for the lens ring, which may induce subconjunctival hemorrhage, are not necessary.

However, corneal surface dehydration and lens condensation could significantly decrease the visibility of the fundus image, and this requires occasional irrigation of the corneal surface or cleaning of the front lens [12, 13]. With BIOM (Oculus Surgical, Inc), Merlin (Volk Optical, Inc.) and OFFISS (Topcon Medical Inc.) noncontact WAVS, focus is achieved by moving entire lens support system up and down to adjust the distance between the cornea and front lens. With Resight noncontact sutureless WAVS, focusing is done internally, using a vario lens system. The distance between the wide-angle lens and the cornea remains constant. Through the 60D lens, epiretinal membrane removal can be done clearly.

Owing to this, it was a common assumption that the operation time of sutureless MIVS using noncontact sutureless WAVS is not decreased despite the high anatomical success rate reported (between 74 and 92.9%) [7, 8]. There have been no previous studies describing the operation time of transconjunctival sutureless MIVS using Resight noncontact sutureless WAVS. We report the average operation time to be 50.55 min, and anticipate this to be used as a data of comparison between different kinds of viewing systems.

Furthermore, we noted that the operation time was not influenced by the preoperative factors of patients, including age, lens status, and macula status. In other words, MIVS can be performed smoothly and widely using a Resight noncontact sutureless WAVS even if the patient has a mild to moderate cataract or intraocular lens. The presence of macular detachment, i.e., the arrangement of subretinal fluid that needs to be drained, did not affect the operation time. However, the relationship of age and operation time calls for careful explanation because of the trend noted (*p* = 0.051). In our study, operation times were longer for patients younger than 50 years old, though not significantly. This may be a result of the additional time spent to induce posterior vitreous detachment in younger patients.

Cryopexy and multiple breaks were only two significant factors which influenced operation time, confirmed by multivariate analysis. The use of adjuvant transconjunctival cryopexy increased operation time significantly (*p* < 0.05), because cryopexy was performed in extremely peripheral retinal breaks in which attempts with focal laser coagulation were tried but ceased in order to avoid touching the lens with the laser probe. RRD with multiple breaks also increased the operation time significantly (p < 0.05), as more laser spots were applied around the breaks.

The single-operation success rate in this study was 93.6%, which is comparable to rates in previous reports of either contact or noncontact WAVS. In our experience, MIVS using noncontact sutureless WAVS is a surgical procedure with consistent anatomical outcomes independent of preoperative variables and surgical factors, including age, BCVA, IOP, refraction, lens status, macular status, the use of adjuvant cryopexy, the type of gas tamponade used, and the operation time. Further analysis of recurrent cases is limited due to small case numbers. Nevertheless, among the 7 eyes with recurrent RRD, 5 eyes had more than 2 breaks (up to 8 breaks) and at least one break located inferiorly.

Multivariate logistic regression model revealed the pre-operation VA is the only factor might predict the improvement of VA compared to pre-operative level. Because there is the best level, 20/20, about the visual acuity, patients with poor pre-operation VA have more possibility of VA improvement. Therefore, PPV with WAVS is still worth to perform on patients with poor VA and macula-off status although final BCVA may not good.

Focusing on the patients who were pseudophakic before MIVS and who underwent cataract operation after MIVS, final BCVA was significantly superior in the pre-MIVS macula-on group and was proportional with shorter operation time. That patients with macula-on RRD will have better visual outcomes is a well-documented event [14–17], and our study supports this observation. That a shorter operation time is correlated with less tissue damage, sooner recovery time, and better outcomes, is another observation in our daily clinical practice. However, to our best knowledge, this is the first study to quantify the relationship between operation time and final BCVA. Even though sutureless transconjunctival MIVS using noncontact sutureless WAVs is a surgery involving very small wounds and without contact with the cornea, there are still possible factors that generate stress to intraocular tissue. The illuminator may cause phototoxicity to the photoreceptor and retinal pigment epithelium in the macula area [18]. Lower temperatures of infusion fluid potentially exert unknown effect on cell metabolism [19]. The turbulent flow of the infusion tube could induce direct mechanical trauma to the retinal tissue [20]. Shorter operation time minimized these retinal damage-causing factors and presumably improved visual outcome. Our study offered evidence of the clinical experiences of a medical center in Taiwan.

There are several limitations to this study. This study was retrospective and the sample size was relatively small. There was no control group of contact WAVS for the operation time because Dr. CJ Lin only uses non-contact sutureless WAVS at China Medical University Hospital. Nevertheless, the comparison between preoperative and intraoperative variables was made and showed significant results.

## Conclusions

Although these factors limit the strength of our conclusions, this study nonetheless demonstrates that sutureless MIVS using the Lumera and Resight sutureless noncontact WAVS is an effective method for primary repair of RRD. The single-operation anatomical success rate is 93.6%, comparable to those of previous studies. BCVA improved significantly after MIVS. The mean operation time is 50.55 min, and is prolonged when adjuvant cryopexy is performed or in cases of RRD with multiple breaks. Finally, shorter operation time and pre-MIVS macula-on status are associated with better final visual outcomes.

## Data Availability

All data generated or analyzed during this study are included in this published article.

## Abbreviations

BCVA: Best-corrected visual acuity
C3F8: Perfluoropropane
IOP: Intraocular pressure
MIVS: Micro-incision vitrectomy surgery
PPV: Pars plana vitrectomy
RRD: Rhegmatogenous retinal detachment
SF6: Sulfur hexafluoride
WAVS: Wide angle viewing systems
